# Linking biogeography to physiology: Evolutionary and acclimatory adjustments of thermal limits

**DOI:** 10.1186/1742-9994-2-1

**Published:** 2005-01-17

**Authors:** George N Somero

**Affiliations:** 1 Department of Biological Sciences, Hopkins Marine Station, Stanford University, Pacific Grove, CA 93950-3094 USA

## Abstract

Temperature-adaptive physiological variation plays important roles in latitudinal biogeographic patterning and in setting vertical distributions along subtidal-to-intertidal gradients in coastal marine ecosystems. Comparisons of congeneric marine invertebrates reveal that the most warm-adapted species may live closer to their thermal tolerance limits and have lower abilities to increase heat tolerance through acclimation than more cold-adapted species. In crabs and snails, heart function may be of critical importance in establishing thermal tolerance limits. Temperature-mediated shifts in gene expression may be critical in thermal acclimation. Transcriptional changes, monitored using cDNA microarrays, have been shown to differ between steady-state thermal acclimation and diurnal temperature cycling in a eurythermal teleost fish (*Austrofundulus limnaeus*). In stenothermal Antarctic notothenioid fish, losses in capacity for temperature-mediated gene expression, including the absence of a heat-shock response, may reduce the abilities of these species to acclimate to increased temperatures. Differences among species in thermal tolerance limits and in the capacities to adjust these limits may determine how organisms are affected by climate change.

## Review

### Introduction

Understanding the roles played by physiological adaptation to temperature in governing the distribution patterns of species has taken on a new urgency because of the potential effects of global warming on both aquatic and terrestrial ecosystems. There is already compelling evidence for widespread changes in ecosystems due to climate change. Recent meta-analyses have shown that species' distribution patterns, the structures of ecosystems, and the timing of annual events (phenology) such as migration and reproduction have changed markedly during the past century, when average global temperatures rose by approximately 0.6°C [1-4]. If the consensus view that average global temperatures will rise by approximately 3°C during this century is correct [5], then even more extensive changes in the biosphere are certain to occur and, in view of this five-fold increase in warming rate, to occur rapidly. Suffice it to say that analyses that can assist in predicting – and, perhaps, even in ameliorating – these biological effects are strongly needed at this time. Because most previous analyses of the biological effects of climate change have been correlative, rather than focused on the underlying causal mechanisms behind the observed effects, there is clearly a need for physiologists to contribute to this important, on-going discussion.

In this brief review, I discuss studies performed with a taxonomically varied group of aquatic ectotherms that shed light on several questions related to the roles played by physiological adaptations in setting species' distribution patterns. The more clearly we understand the mechanistic basis of biogeography, the better prepared we will be to predict the effects of climate change on distribution patterns and ecosystem structure. The questions I address include the following. First, how do thermal tolerance limits differ among species adapted to different temperatures? Which species are most likely to be threatened by increases in habitat temperatures, i.e., which species currently live closest to their thermal tolerance limits? Second, what are the "weak links" in physiological systems that appear most likely to set thermal tolerance limits? Can we identify physiological, biochemical, and molecular level effects that account for thermal tolerance ranges? Third, how do capacities for acclimation to changes in habitat temperature differ among species? Which species have the greatest – or the least – ability to acclimate to increases in temperature? Mechanistically speaking, what types of acclimatory changes are needed to adapt physiological systems to permit tolerance of new thermal conditions? Fourth, what types of changes in gene expression are needed to achieve thermal acclimation? How does gene expression during acclimation to rapid, diurnal change in temperature differ from the response to slower, seasonal time-scale change in temperature? How do stenothermal and eurythermal species differ in their capacities for altering gene expression in temperature-adaptive manners? Have extreme stenotherms like Antarctic animals that have evolved for many millions of years under highly stable, cold temperatures lost key abilities to acclimate to increasing temperatures?

To address these questions, I focus primarily on studies that our laboratory has performed on aquatic ectothermic species that have been chosen for study because they seemed to fit well the August Krogh Principle, which can be paraphrased as follows: For any question a biologist asks, Nature can provide a most appropriate study organism. Through study of such "Kroghian" species, it has been possible to obtain at least initial answers to the several questions raised above.

### Thermal tolerance relationships of porcelain crabs (genus *Petrolisthes*) and turban snails (genus *Tegula*)

One of the challenges facing a comparative or evolutionary physiologist wishing to elucidate adaptive differences among species is the need to separate effects due to phylogeny from true adaptive variation. One means of achieving this end is to study groups of closely related organisms from widely different habitat conditions, for which a comprehensive phylogeny exists. A group of species that is especially "Kroghian" from this standpoint are porcelain crabs (genus *Petrolisthes*) from the eastern Pacific Ocean [6]. Congeners of *Petrolisthes *are abundant (46 species occur in the eastern Pacific), found over wide ranges of latitude, and occur in subtidal and intertidal habitats. Among these congeners, body temperatures extend from slightly below 0°C to over 40°C; for some eurythermal species, the range of body temperatures across different seasons can be over 32°C [7-10].

**Figure 1:**
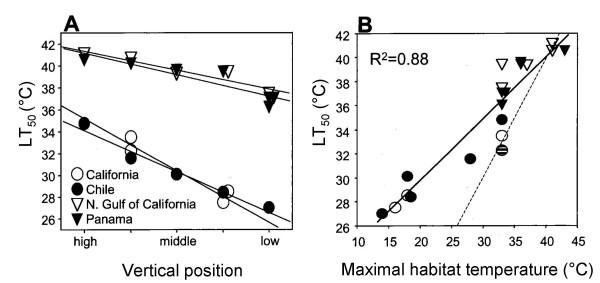
Heat tolerance of porcelain crabs. Upper thermal tolerance limits (LT_50 _(°C)) of congeneric porcelain crabs (genus *Petrolisthes*) native to eastern Pacific habitats in California, Chile, the northern Gulf of California, and Panama and occurring at different heights along the subtidal-to-intertidal gradient. Each symbol represents a different species of crab. B. Thermal tolerance *versus *maximal habitat temperature, with a line of unity given to show the proximity of current habitat temperatures to lethal temperatures. Hatched symbol represents *P. cinctipes*. The R^2 ^value for the regression line for all species is given. Data are from [10].

The thermal tolerance limits of field-acclimatized temperate (California and Chile), subtropical (northern Gulf of California) and tropical (Panama) congeners of *Petrolisthes *from different heights along the subtidal to intertidal gradient are shown in Fig. 1. Data are presented as LT_50 _values, the temperatures at which fifty percent of an experimental population is killed by the heat treatment. Heating rates simulated measured rates of environmental temperature change found in the species' habitats [10]. The predicted correlation between adaptation temperature and LT_50 _is found (Fig. 1A): the most heat-tolerant intertidal species, those from subtropical and tropical habitats, had LT_50_s that were approximately 15°C higher than those of temperate sub-tidal species and 6–7°C higher than temperate intertidal species. However, the likelihood of heat death facing the different species under natural habitat conditions is not reflected by the LT_50 _*per se*, but rather the by proximity of this trait to current extremes of habitat temperature. As shown by the alignment of LT_50 _values *vis à vis *the line of unity (LT_50 _= maximal habitat temperature) in Fig. 1B, warm-adapted intertidal species are in much greater jeopardy from heat death than their more cold-adapted subtidal relatives. Moreover, the abilities of congeners of *Petrolisthes *to increase thermal tolerance through acclimation to increased temperature are less in intertidal species than in more cold-adapted subtidal congeners [10]. Thus, warm-adapted intertidal species face greater current – and, most likely, future – threats from high temperatures than less heat-tolerant, subtidal congeners.

**Figure 2:**
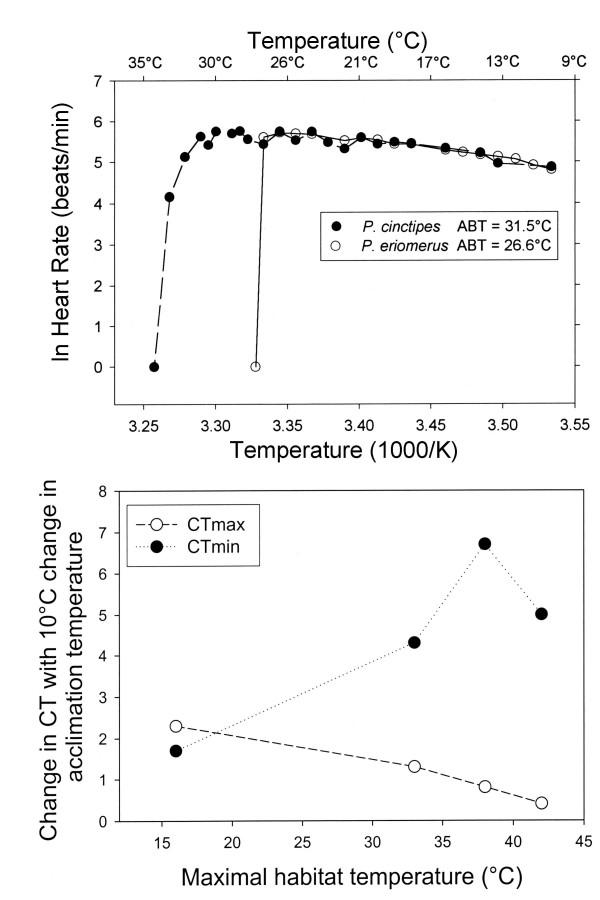
The effects of temperature on cardiac activity in porcelain crabs. Upper panel. Arrhenius plots of ln heart rate (beats per minute) *versus *measurement temperature (1/K) for two congeners of porcelain crabs, *Petrolisthes cinctipes *and *P. eriomerus*, having maximal habitat temperatures of approximately 32°C and 16°C, respectively. Arrhenius break temperatures (ABTs) are the temperatures at which a sharp decrease in heart rate is noted (see Stillman & Somero [9] for computational methods). Lower panel. Acclimatory-induced change in upper and lower critical temperatures (CT_max _and CT_min_, respectively) of heart function for 4 congeners of *Petrolisthes*: *P. cinctipes *and *P. eriomerus *from temperate central California habitats, and *P. gracilis *and *P. hirtipes *from the northern Gulf of California. CT_max _is the ABT, and CT_min _is the temperature at which heart beat ceased as temperature was lowered. Decreasing temperatures did not cause a sharp break in Arrhenius plots, so ABTs could not be determined at low temperatures [8,9]. Acclimation temperatures were 8°C and 18°C for the California species and 15°C and 25°C for the Gulf of California species. The differences in CT_max _and CT_min _between the two acclimation groups of each species are shown. Each symbol represents a different species, whose maximal habitat temperature is given on the abscissa. Figure modified after [8].

One physiological system that is a "weak link" in the thermal tolerance of these species is heart function. As shown in Fig. 2A, sharp reductions in heart rate occur when a species-specific high temperature is reached. This is termed the "Arrhenius break temperature" (ABT) to denote that it is the temperature at which a sharp discontinuity in the slope of an Arrhenius plot (ln rate of heart beat *versus *reciprocal temperature (K)) occurs. Once the ABT is exceeded, heart function of porcelain crabs does not recover from heat stress. ABT values for the temperate congeners *P. cinctipes *(intertidal) and *P. eriomerus *(subtidal) are 31.5°C and 26.5°C, respectively. The maximal habitat temperatures for the two species are approximately 31°C and 16°C, respectively [9,10]. Thus, whereas the subtidal congener has an approximately 10°C range between its highest habitat temperature and the upper thermal limit of heart function, the intertidal congener's upper habitat temperature, LT_50_, and ABT for heart function are essentially the same, 31–32°C. It bears emphasizing that ABT values for other physiological processes, for example, mitochondrial respiration [11,12] and enzymatic activity [13], may be considerably higher than upper lethal temperatures of the whole organism. For some species of animals, then, heart function appears to be a "weak link" in the "chain" of physiological processes that govern thermal tolerance.

The acclimatory plasticity of ABTs of heart function differs among species in a parallel fashion to acclimatory change in LT_50 _(Fig. 2B[8]). In this experiment, 4 congeners were studied: the two temperate species from California discussed above, *P. cinctipes *and *P. eriomerus*, and upper intertidal and mid-intertidal species from the northern Gulf of California, *P. gracilis *and *P. hirtipes*, respectively. Acclimation temperatures were 8°C and 18°C for the temperate species and 15°C and 25°C for the sub-tropical species. ABT (= CT_max_) of *P. eriomerus *rose by 2.3°C when acclimation temperature was increased by 10°C, but the most warm-adapted species, *P. gracilis*, increased ABT (CT_max_) by only 0.5°C. The eurythermal intertidal species did exhibit greater capacities than the subtidal species to acclimate to lower temperatures, however, as shown by their greater ability to lower the temperature at which heart beat ceased (CT_min_) (Fig. 2B). Despite being eurythermal, the warm-adapted species are again seen to be in greatest jeopardy from further increases in maximal habitat temperature; their abilities to extend their tolerance of lower temperatures is not matched by a similar capacity for increasing tolerance of heat.

The differences in thermal tolerance and capacities for warm acclimation noted with porcelain crabs were mirrored in studies done with another set of congeneric marine invertebrates found at different vertical positions along the subtidal to intertidal gradient, turban snails of the genus *Tegula *[14]. Intertidal and subtidal *Tegula *congeners exhibit significant differences in thermal limits of protein synthesis and onset temperatures for production of heat-shock proteins that reflect their vertical distributions [15,16]. In agreement with these data, field-acclimatized populations of three *Tegula *congeners had significantly different thermal limits of heart function: the low- to mid-intertidal congener, *T. funebralis*, had a higher ABT of heart function (31.0°C) than two subtidal congeners, *T. brunnea *and *T. montereyi *(ABTs of 25.0°C and 24.2°C, respectively) [17]. The ABT of *T. funebralis *approximated the highest body temperature recorded for this species in the field, 32°C [15]. Body temperatures for the subtidal species only rarely reach 20°C [15], so these species would seldom, if ever, experience heart failure in their habitats. Furthermore, in agreement with the studies of porcelain crabs [8], *T. funebralis *had a lower ability to increase ABT during warm acclimation than the subtidal species [17].

These interspecific differences in thermal tolerance limits for whole organism survival and maintenance of heart function provide important lessons concerning biogeographic and vertical patterning and the potential effects of climate change. First, the differences in thermal tolerance between subtidal and intertidal species indicate that the former species would be unable to persist at the upper habitat temperatures commonly found in the intertidal zone during daytime low tides in warm seasons. For example, the temperate subtidal species *Petrolisthes eriomerus *has an upper lethal temperature approximately 6°C below the upper limit of body temperature recorded for the intertidal species *P. cinctipes*. Interspecific variations in LT_50 _and ABT values also mirror the biogeographic patterning found across latitude. For instance, north temperate congeners of *Petrolisthes *would be unable to survive under the habitat conditions found in the northern Gulf of California or Panama. The close agreement among LT_50_, ABT, and maximal habitat temperatures for intertidal species suggests that further increases in habitat temperature could have strong impacts on the persistence of these species in their habitats, especially when the limits of acclimatory ability are reached. Because the greatest thermal stress occurs during emersion periods during the warmest time of the day, and because solar heating is more critical than ambient water temperature, the precise consequences of global warming are difficult to predict. Furthermore, because of latitudinal variation in the timing of tidal cycles, these and other intertidal species may face greater threats from climate change at mid-latitudes than at lower latitudes [18]. Despite these uncertainties, however, the seemingly paradoxical conclusion reached above, to the effect that many warm-adapted species are more threatened by increases in habitat temperature than cold-adapted congeners, appears valid and should be taken as a caveat that predicting the consequences of climate change is a complex challenge.

### Acclimatory changes in gene expression: eurytherms *versus *stenotherms

The abilities of ectotherms to cope satisfactorily with increases in habitat temperature are based on several factors, including the proximity of habitat temperatures to the edges of the thermal tolerance range and the abilities to shift the tolerance range through acclimatization. In the case of porcelain crabs and turban snails, acclimatory ability was shown to vary among species. This finding that even closely related congeneric species differ in acclimatory ability raises questions about the mechanistic basis of phenotypic plasticity and the roles of evolutionary thermal history in establishing this plasticity.

The ability to modify the phenotype in response to a change in body temperature is certain to depend strongly on a capacity for modulating gene expression in an adaptive manner. Although comprehensive surveys of temperature-induced shifts in gene expression have not been done for any marine organisms, studies of model species, defined here as species for which the genome has been sequenced and relatively well annotated, are beginning to reveal the pervasiveness of stress-induced shifts in gene expression. The studies of Gasch and colleagues [19] on yeast, for example, have shown that a variety of physical and chemical stressors, including temperature, hypoxia, reactive oxygen species and osmotic shock, triggered relatively similar changes in expression of approximately 900 genes, which represents approximately 14% of the yeast genome. They termed this gene regulatory response the "environmental stress response (ESR)". Because the proteins, lipids and nucleic acids that form the structural foundation of all physiological processes of cells will be affected in a qualitatively similar manner by changes of temperature in all organisms [20], it is reasonable to conjecture that an ESR generally similar to that found in yeast may characterize all species. For instance, induction of stress-induced chaperones (heat-shock proteins) for repair of damaged proteins, and shifts in synthesis of the various enzymes required for modifying the properties of lipid-based systems are likely to be ubiquitous events in the thermally induced ESRs of different species [20].

Despite the likely occurrence among species of generally similar requirements for temperature-adaptive shifts in gene expression, species that fall into different regions of the stenotherm to eurytherm spectrum may have distinctly different capacities for acclimatory regulation of transcription. In the context of effects of climate change, one of the most important interspecific differences relates to the ability of extreme stenotherms, which may have evolved for millions of years under stable thermal conditions, to alter gene expression in the face of temperature change. The Antarctic notothenioid fishes are a primary case in point. These species have evolved for 10–14 million years in a thermally stable "ice bath," in which annual temperature variation is usually less than 1–2°C [21]. McMurdo Sound populations of notothenioids are unable to acclimate to temperatures above approximately 4°C [22,23], making these species among the most stenothermal of organisms. Antarctic marine invertebrates, too, are extreme stenotherms, with heat death occurring at temperatures only a few degrees above 0°C [24]. In the case of the notothenioid fish *Trematomus bernacchii*, the lack of capacity to acclimate to elevated temperatures may stem in part from deficiencies in its ability to alter gene expression as its body temperature changes. Unlike virtually all other species, *T. bernacchii *is unable to increase the synthesis of any class of heat-shock protein following thermal stress [23]. Lacking this ability, *T. bernacchii *may be unable to effect adequate chaperone-mediated restoration of the native structures of heat-denatured proteins. A build-up of aggregates of denatured proteins is likely to be cytotoxic and lead to the eventual death of cells [25]. The absence of a heat-shock response in Antarctic notothenioids, but its presence in temperate New Zealand notothenioids [26], suggests that evolution under cold, stable thermal conditions has led to a depletion of the genetic resources of Antarctic fish. The inability of some notothenioid species to synthesize hemoglobins [27] or myoglobins [28] is another illustration of the depauperate genome of these cold-adapted stenotherms. Loss of oxygen transport pigments is viewed as a reflection of the lack of need for these pigments because of the combination of high oxygen solubility at low temperatures and the generally sluggish locomotory habits of the notothenioids [27,28]. In general, disappearance of these diverse genetic capacities in notothenioids is consistent with relaxed selection against the loss of genes that are no longer needed under conditions of stable low temperatures. Although future work on temperature-induced changes in the transcriptomes of Antarctic stenotherms will be needed before any broad generalizations can be made about the contents of their "genetic tool kits," it seems likely that the stenothermy of these species could be due in large measure to a loss of the type of acclimatory plasticity that is found in eurytherms. Lacking this phenotypic plasticity, Antarctic stenotherms seem uniquely vulnerable to the effects of global warming.

Even among eurythermal ectotherms, there may be a variety of capacities for modulating gene expression to compensate for changes in body temperature. Although few data are currently available to test this point, acclimatory responses to rapid, diurnal variations in body temperature may require different shifts in gene expression from those that characterize slower, e.g., seasonal time-scale, responses to temperature change. A recent study [29] of temperature-induced changes in the transcriptome (the population of messenger RNA (mRNA) molecules in the cell) of liver tissue of a eurythermal teleost fish, *Austrofundulus limnaeus*, revealed wide-scale shifts in gene expression, as noted earlier for yeast [19]. A total of 540 mRNAs out of 4,992 examined changed by two-fold or more during acclimation. Many of the mRNAs changing in response to thermal acclimation were for proteins that are well known to be key elements of acclimatory response. For example, transcripts for heat-shock proteins and for enzymes involved in temperature-compensatory shifts in membrane lipid composition showed the predicted changes. However, for both classes of proteins, distinctly different expression profiles were found under the two acclimation regimes employed in this study: (i) steady-state acclimation at 20°C or 37°C for up to two weeks, and (ii) diurnally cycling temperatures that varied from a high of 37°C near mid-day to 20°C at night, a cycle that simulates the environmental conditions the species encounters in its shallow pond habitats in South America [29]. Under steady-state acclimation, transcripts for heat-shock proteins Hsp70 and Hsp90 showed the largest amount of change among molecular chaperones. Under cycling conditions, low-molecular-mass chaperones showed the greatest change. This finding suggests that protein damage and the repair costs it entails may differ under constant *versus *intermittent heat stress. Although both acclimation regimes led to changes in transcript abundance for proteins associated with alterations in lipid composition, differences between cycling and steady-state acclimation were noted in the types of lipids that appear to be produced. For steady-state acclimation, the expected changes in transcripts for enzymes involved in acyl chain double bond content (saturation) were observed, consistent with many previous studies of homeoviscous acclimation in membranes [20]. During cycling conditions, changes in transcripts for proteins involved in cholesterol biosynthesis and transport were more pronounced than shifts in mRNAs for enzymes of acyl chain biosynthesis. Although the significance of this difference in adaptive response is not known, it seems possible that insertion into or removal from the membrane of cholesterol could be achieved more rapidly than changes in the composition of phospholipids. In any event, differences in the time-frame of thermal stress may lead to differences in the types of temperature-compensatory strategies used to modulate the fluidity and phase of membranes.

**Figure 3:**
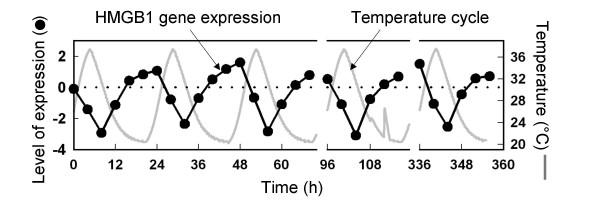
Temperature effects on the transcriptome of the eurythermal fish *Austrofundulus limnaeus*. Changes in liver tissue in the level of the mRNA encoding high mobility group B1 protein (HMGB1), during thermal cycling between 37°C and 20°C. The Y-axis plots the logarithm of the ratio of expression in experimental (thermally cycled) *versus *control animals. Expression of the *hmgb1 *gene showed no circadian rhythm in fish held at constant temperature. Data from [29].

One of the most striking changes in transcript abundance observed in the study of *Austrofundulus limnaeus *was for the message encoding high mobility group b1 protein (HMGB1)(Fig. 3). High mobility group proteins are DNA-binding proteins that exert wide-ranging effects on transcriptional activity [30]. Rather than serving as transcriptional regulators for specific genes, HMGB1 proteins are general activators of transcription that exert their effects by influencing the transcriptional competence or "openness" of DNA structure. Increases in concentrations of HMGB1 favor open DNA structures and increased transcriptional activity of many genes. The changes in mRNA for HMGB1 found during thermal cycling (Fig. 3) and during steady-state acclimation [29] indicate an extremely tight control of this message. The increase in content of HMGB1 message with falling temperature (steady-state or cycling) is consistent with the role of this protein in maintaining DNA structure in an open, transcriptionally competent state. Thus, decreases in temperature will enhance the stability of the non-covalent bonds that stabilize higher order of DNA structure. To offset this increased stability of DNA structure, the cell may increase the concentrations of HMGB1 proteins, thereby allowing transcriptional activity to be temperature-independent. This is not to suggest that changes in HMGB1 protein will lead to genome-wide changes in transcriptional activity, of course. Rather, by increasing the openness of DNA structure, the promoter regions of genes will be susceptible to the effects of gene-specific transcription factors whose influences will lead to temperature-specific alterations in the transcriptome.

The changes in gene expression noted in *Austrofundulus limnaeus *and in a recent study of carp [31] represent an initial view of the complexity of transcriptional changes that ectothermic animals may experience during thermal acclimation, either to short-term or long-term changes in body temperature. Different changes to the transcriptome may be involved in different time courses of acclimation. It will be important to establish whether extreme stenotherms like Antarctic fish are capable of adjusting their transcriptomes in temperature-adaptive manners, when body temperature is increased. The importance of maintaining DNA structure in an open configuration that allows transcription factors to effectively modulate gene expression is a phenomenon that merits additional study. This capacity may be of pivotal significance in determining the effectiveness with which ectotherms can respond to changes in temperature; it may be foundational for most, if not all, of the temperature acclimation response. How this capacity differs between eurytherms and stenotherms may determine how these two groups cope with thermal fluctuations in their present habitats and what their potentials for coping with climate change are likely to be.

## Conclusions

Ectothermic species differ widely in thermal tolerance limits and in their abilities to adjust these limits in temperature-adaptive manners. Comparisons of congeneric species from different latitudes and different positions along the subtidal to intertidal gradient have provided important insights into adaptive variation and the threats posed by increased habitat temperature. In what at first view may seem paradoxical, warm-adapted congeners may be more threatened by increased temperatures than their cold-adapted subtidal relatives. This difference stems from two factors: the proximity of current habitat temperatures to upper lethal temperatures (LT_50_s) and the more limited abilities to acclimate to increases in temperature noted for intertidal species. The physiological determinants of upper thermal limits are certain to be multifarious, but heart function stands out as a key contributor to these limits. In porcelain crabs and turban snails, upper lethal temperatures coincide closely with temperatures of heart failure. Such cardiac effects also could contribute to a shift from aerobic to anaerobic processes of ATP generation, which has been proposed as one important mechanism of death from high and low temperatures [32,33]. Acclimatory response to change in body temperature is likely to be an important determinant of the effects of global warming on species. Temperature-adaptive shifts in gene expression serve as a foundation for physiological acclimation. Changes in the transcriptome differ between long-term, steady-state acclimation and responses to diurnally cycling temperatures. The ability to modulate gene expression requires an openness of DNA structure, which appears to be closely regulated during steady-state and cycling thermal regimes. Being able to modulate the transcriptional competence of DNA in the face of changing temperature, to ensure that necessary shifts in gene expression can occur, may be a fundamental requirement for thermal acclimation. Species such as Antarctic notothenioid fishes that have had a long evolutionary history at constant temperatures may have lost many of the critical gene regulatory responses needed for thermal acclimation and, as a consequence, they may be uniquely vulnerable to global warming.
